# Visualizing the geography of genetic variants

**DOI:** 10.1093/bioinformatics/btw643

**Published:** 2016-11-21

**Authors:** Joseph H Marcus, John Novembre

**Affiliations:** 1 Department of Human Genetics, University of Chicago, Chicago, USA; 2 Department of Ecology and Evolution, University of Chicago, Chicago, USA

## Abstract

**Summary:**

One of the key characteristics of any genetic variant is its geographic distribution. The geographic distribution can shed light on where an allele first arose, what populations it has spread to, and in turn on how migration, genetic drift, and natural selection have acted. The geographic distribution of a genetic variant can also be of great utility for medical/clinical geneticists and collectively many genetic variants can reveal population structure. Here we develop an interactive visualization tool for rapidly displaying the geographic distribution of genetic variants. Through a REST API and dynamic front-end, the *Geography of Genetic Variants (GGV)* browser (http://popgen.uchicago.edu/ggv/) provides maps of allele frequencies in populations distributed across the globe.

**Availability and Implementation:**

GGV is implemented as a website (http://popgen.uchicago.edu/ggv/) which employs an API to access frequency data (http://popgen.uchicago.edu/freq_api/). Python and javascript source code for the website and the API are available at: http://github.com/NovembreLab/ggv/ and http://github.com/NovembreLab/ggv-api/.

**Supplementary information:**

Supplementary data are available at *Bioinformatics* online.

## 1 Introduction

Genetics researchers often face the problem that they have identified one or many genetic variants of interest using an approach such as a genome-wide association study and then would like to know the geographic distribution of the variant. For example, the researcher may hope to address: (i) implications for genomic medicine (e.g. Is a risk allele geographically localized to a certain patient population? What population should be studied to observe variant carriers? (Rosenberg *et al.*, 2010)); or (ii) the evolutionary history of the variant in question (e.g. does the variant correlate with a known environmental factor in a manner suggestive of some geographically localized selection pressure? (Novembre and Di Rienzo, 2009)). A simple geographic map of the distribution of a genetic variant can be incredibly insightful for these questions.

Contemporary population genetics researchers are also faced with the challenge of large, high-dimensional datasets. For example, it is not uncommon for a researcher in human genetics to have a dataset comprised of thousands of individuals measured at hundreds of thousands or even millions of single nucleotide variants (SNVs). One common approach to visualizing such high-dimensional data is to compress the SNV dimensions down to a small number of latent factors, using a method such as principal components analysis (Patterson *et al.*, 2006), or a model-based clustering method such as STRUCTURE (Pritchard *et al.*, 2000). While these methods are extremely valuable, researchers can use them too often without inspecting the underlying variant data in more detail. A natural approach to gaining more insight to the overall structure of a population genetic dataset is to visually inspect what geographic patterns arise in allele frequency maps.

Unfortunately, generating geographic allele frequency maps is time-consuming for the average researcher as it requires a combination of data-wrangling methods (Kandel *et al.*, 2011) and map-making techniques that are unfamiliar to most. Our aim here is to produce a tailored system for rapidly constructing informative geographic maps of allele frequency variation.

Our work is inspired by past tools such as the ALFRED database (Rajeevan *et al.*, 2012) and the maps available on the HGDP Selection browser (Pickrell *et al.*, 2009) whose allele frequency output and plots have been used in research articles (e.g. Coop et al., 2009; Pickrell *et al.*, 2009), books (e.g. Dudley and Karczewski, 2013), and have been made available on the UCSC Genome Browser (available under the HGDP Allele Freq track of the browser, Kent *et al.*, 2002).

Taking advantage of recent advances in web-based visualization tools (Bostock *et al.*, 2011), we aim to address the significant visualization challenges that are inherit in the production of geographic allele frequency maps for large population genomic datasets, including dynamic interaction, display of rare genetic variation, and representation of uncertainty in estimated allele frequencies due to variable sample sizes.

## 2 Approach

The Geography of Genetic Variants browser (GGV) uses the scalable vector graphics and mapping utilities of D3.js (Bostock *et al.*, 2011). The front-end provides legends for the map and various configuration boxes to allow users to query different datasets or choose visualization options.

**Fig. 1.**
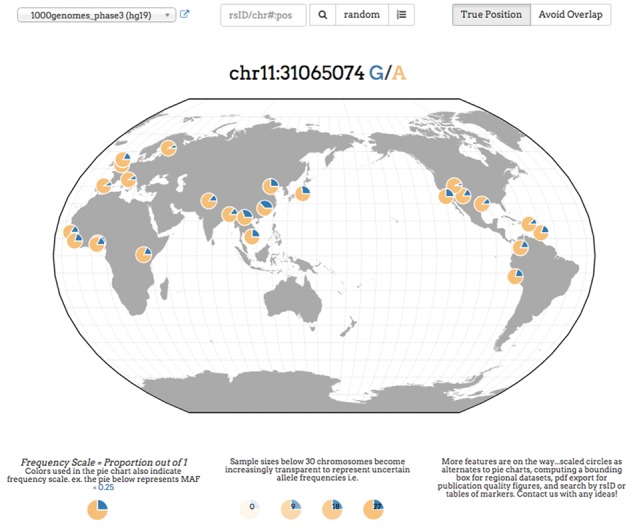
Example screenshot from the Geography of Genetic Variants browser using The 1000 Genomes Project Consortium (2015) data. Each pie chart represents a population with the blue slice of the pie displaying the frequency of the global minor allele

In order to allow for easy access to commonly used public genomic datasets, such as the 1000 Genomes project (The 1000 Genomes Project Consortium, 2015) or Human Genome Diversity project (Li *et al.*, 2008), we have developed a REST API (Grinberg, 2014). The API allows retreival of SNVs by position, rsid (Sherry *et al.*, 2001) or at random. After a query, the GGV displays the allele frequencies as a collection of pie charts where each represents the frequency of the globally minor allele in a single population (Fig. 1).

We implement a variety of features: **(1) Rare variants.** Many alleles are rare (e.g. The 1000 Genomes Project Consortium, 2015), and displaying them can be a challenge with proportional scales that range from zero to one. To address this challenge we re-scale frequencies, so that small frequencies become visible. Specifically, we use a frequency scale that is indicated in a legend below the map and represented by varying color in the pie charts (Fig. 1, Supplementary Fig. S1, Supplementary Table S1). **(2) Uncertainty in frequency data.** We use varying transparency in a population’s pie chart: estimated frequencies with higher levels of sampling error (e.g. those from samples with *n* < 30) are made more transparent, and hence less visible, on the map (Fig. 1, Supplementary Fig. S2). **(3) Overlapping populations.** We use force-directed layouts of the populations such that no two points are overlapping each other, and yet the points will be pulled towards their true origins (Fig. 1, Supplementary Fig. S3). Also, by hovering the mouse cursor over any population, a user can see the population labels and precise frequency information.

By allowing rapid generation of allele frequency maps, we hope to facilitate the interpretation of variant function and history by practicing geneticists. Also, for students of human diversity, it is often difficult to conceptualize classic statements regarding how most variation in humans is shared among populations (Lewontin, 1972) and how the fixation index *F_ST_* is relatively low globally (10–15% The 1000 Genomes Project Consortium, 2015). We hope that the ability to query random variants from major human population genetic samples will allow students to appreciate the structure of human genetic diversity in an approachable and intuitive form.

## Acknowledgements

We acknowledge the Research Computer Center at the University of Chicago, especially Jeff Tharsen and Alex Mueller, for on-going support and development, as well as John Zekos for server administration support and members of the Novembre Lab.

## Funding

Support for this work was provided by the National Institutes of Health via the Big Data to Knowledge initiative (1U01 CA198933-0, JN) and the National Institute of General Medical Sciences under training grant award number T32GM007197 (JHM).

*Conflict of Interest*: none declared.

## Supplementary Material

Supplementary Data
