# Supplementary material for: Visualizing the geography of genetic variants
Source: Bioinformatics. 2016 Nov 21;33(4):594–5. doi: 10.1093/bioinformatics/btw643 (PMC5408806; doi:10.1093/bioinformatics/btw643)
Supplement: Supplementary Data [file btw643_supp.pdf]

Supplementary Information

Visualizing the Geography of Genetic Variants

Joseph H. Marcus*[1] and John Novembre*[1,2]

[1]Department of Human Genetics, University of Chicago, Chicago, IL,USA
[2]Department of Ecology and Evolutionary Biology, University of Chicago, Chicago, IL,USA

Expanded discussion

A major challenge of using a geographic representation of genetic variation in humans is that the samples must be associated with a geographic location. While doing so is generally immensely helpful, it has inherent complexity and limitations. For example, practitioners must make choices regarding representing where an individual was sampled for the study (e.g. the city of a major research center) or choosing a location that is more representative of an individual's ancestral origins (e.g. based on the birthplaces of recent ancestors, such as grandparents). We do not proscribe a general solution to this problem, and for the current defaults we use locations based on the approach taken in the source publications. A future feature will allow alternative location schemas to be used for the populations in a dataset.

We also envision a variety of future extensions to the GGV that would allow for further dissection of geographic structure in large-scale population genomic datasets. Providing an interactive means of browsing neighboring variant sites near a SNP of interest would offer a unique view into patterns of linkage disequilibrium around that focal SNP. This feature would be relevant to both medical geneticists conducting genome-wide association studies with interests in fine mapping as well as population geneticists interested in scanning the genome to detect signatures of positive selection. We imagine that incorporating a chromosomal browser such as jbrowse (Skinner *et al.*, 2009) within the GGV would be greatly utilized by researchers and educators alike.

*Address correspondence to JHM (jhmarcus@uchicago.edu) or JN (jnovembre@uchicago.edu).

Figures and Tables

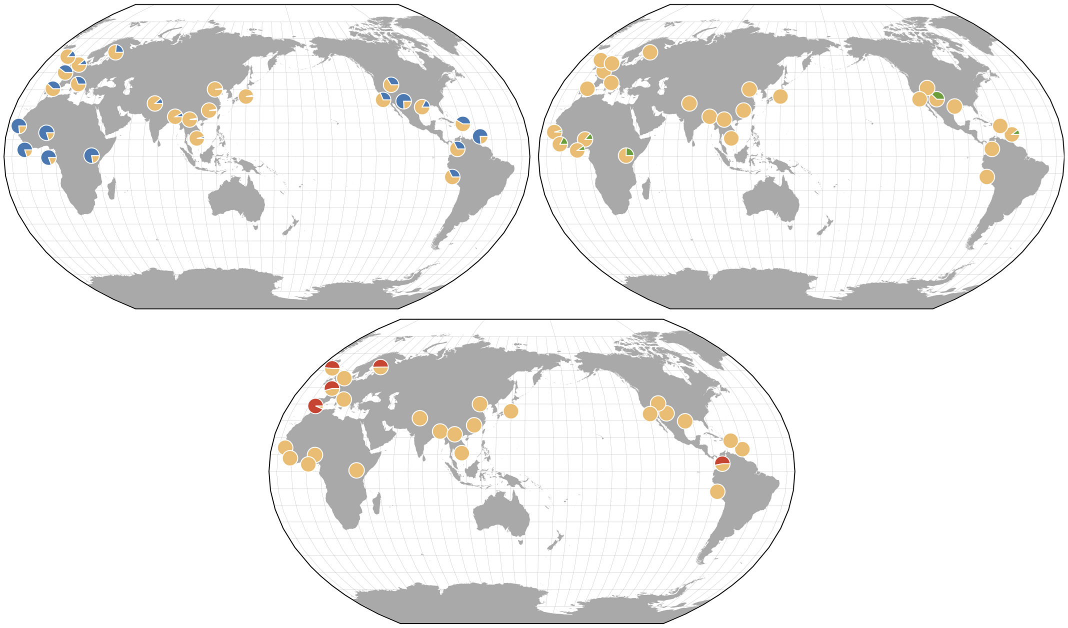

Figure S1: Example maps from the Geography of Genetic Variants browser displaying the use of frequency scales for more expressive representations of rare variation on geographic maps. The blue pie charts convey a given minor allele frequency out of 100 percent, the green out of 1 percent, and the red out of 0.01 percent. The data are from The 1000 Genomes Project Consortium (2015).

**Sample Frequency**	**Display Frequency Scale**	**Displayed Image**
0.25	1	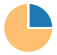
0.025	0.1	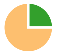
0.0025	0.01	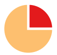
0.00025	0.001	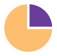

Table S1: Rare variants present a challenge for display. To address this challenge, the GGV browser changes the displayed image and the frequency scale of the map depending on the input sample frequency. As an example, a variant with a frequency 0.0025 is shown as a pie-chart that is 25% full and a frequency scale of 0.01 is marked in the legend of the map.

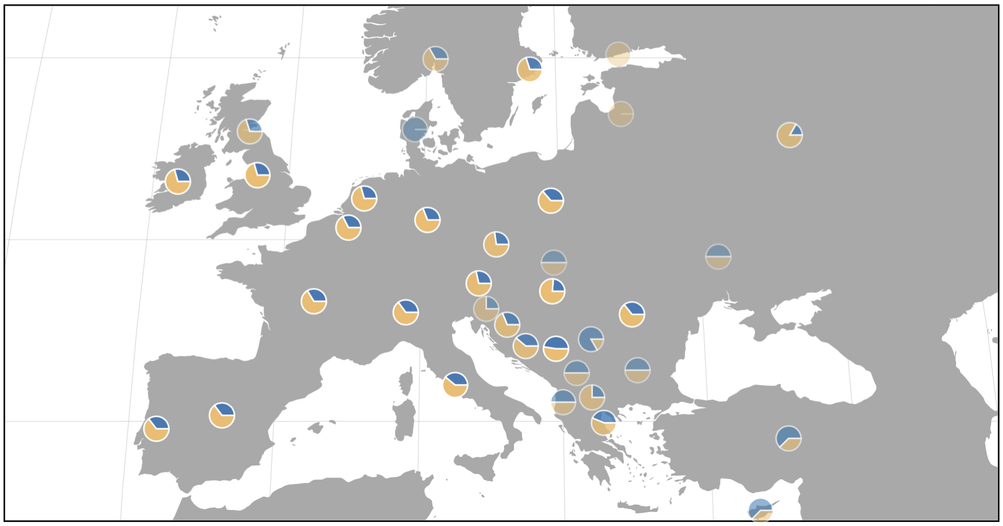

Figure S2: Example map from the Geography of Genetic Variants browser displaying the use of varying transparency of population pie charts to represent uncertainty in allele frequencies. The transparency is scaled in proportion to the number of observed chromosomes in each population for a particular variant. The frequency data and population identifiers are from Novembre *et al.* (2008).

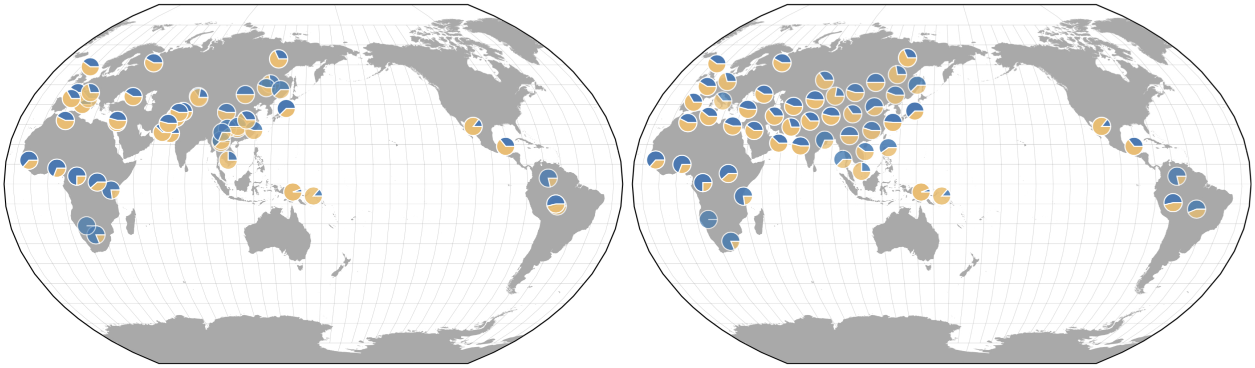

Figure S3: Example maps from the Geography of Genetic Variants browser displaying the use of a force directed layout to limit visual clutter when many populations overlap in geographic position. The left map shows the original population locations while the right shows the application of the force directed layout.

Appendix

Example 1: Query by rsid

```
http://popgen.uchicago.edu/ggv_api/freq_table?data="1000genomes_phase3_table"&rsID=rs1834640
[
  {
    "alleles": ["A", "G"],
    "pos": ["-15.310139", "13.443182"],
    "pop": "GWD",
    "nobs": "226",
    "xobs": "17",
    "freqscale": 1,
    "freq": [0.0752212389381, 0.9247787610619],
    "chrom_pos": "15:48392165",
    "rawfreq": 0.0752212389381
  }, ...
]
```

Example 2: Query by chromosome position

```
http://popgen.uchicago.edu/ggv_api/freq_table?data="1000genomes_phase3_table"&chr=14&pos=37690093
[
  {
    "alleles": ["G", "A"],
    "pos": ["-15.310139", "13.443182"],
    "pop": "GWD",
    "nobs": "226",
    "xobs": "0",
    "freqscale": 0.01,
    "freq": [0.0, 1.0],
    "chrom_pos": "14:37690093",
    "rawfreq": 0.0
  }, ...
]
```

Example 3: Random query

```
http://popgen.uchicago.edu/ggv_api/freq_table?data="1000genomes_phase3_table"&random_snp=True
[
  {
    "alleles": ["T", "C"],
    "pos": ["-15.310139", "13.443182"],
    "pop": "GWD",
    "nobs": "226",
    "xobs": "0",
    "freqscale": 0.01,
    "freq": [0.0, 1.0],
    "chrom_pos": "5:42452893",
    "rawfreq": 0.0
  }, ...
]
```

References

Novembre, J., Johnson, T., Bryc, K., Kutalik, Z., Boyko, A. R., Auton, A., Indap, A., King, K. S., Bergmann, S., Nelson, M. R., *et al.* (2008). Genes mirror geography within Europe. *Nature*, **456**(7218), 98–101.

Skinner, M. E., Uzilov, A. V., Stein, L. D., Mungall, C. J., and Holmes, I. H. (2009). JBrowse: a next-generation genome browser. *Genome Research*, **19**(9), 1630–1638.

The 1000 Genomes Project Consortium (2015). A global reference for human genetic variation. *Nature*, **526**(7571), 68–74.
